# Comparative insecticidal efficacy and biochemical impact of nano-encapsulated citronella and geranium essential oils against *Spodoptera littoralis* (Lepidoptera: Noctuidae)

**DOI:** 10.1038/s41598-026-52470-2

**Published:** 2026-05-19

**Authors:** Enas Adel Abd-Elatef, Abeer Mohammed, Soad Mohamed Osman, Mona N. Wahba, Ahmed A. El-Rashedy, Hanaa E. Sadek

**Affiliations:** 1 Plant Protection Research Institute, Agricultural Research Center ( ARC), Giza, Egypt; 2 Microbial Biotechnology Department, Faculty of Biotechnology, University of Sadat City, Sadat City, Egypt; 3 Department of Medical Laboratory Techniques, Al-Farahidi University, Baghdad, 10021 Iraq; 4 Chemistry of Natural and Microbial Products Department, National Research Center (NRC), Cairo, Egypt; 5 Department Organic and Medicinal Chemistry, Faculty of Pharmacy, University of Sadat City, Menoufia, 32897 Egypt; 6 Department of Pests and Plant Protection, Agricultural and Biological Research Institute, National Research Centre, Cairo, 12622 Egypt

**Keywords:** *Spodoptera littoralis*, Citronella oil, Geranium oil, Toxicity, Bionomics, Enzymatic analyses, Molecular docking, Nano-emulsions, PEG, Biochemistry, Biotechnology, Chemical biology, Microbiology, Plant sciences

## Abstract

This study assessed the insecticidal efficacy of bulk and nano-formulated essential oils of citronella (*Cymbopogon nardus*) and geranium (*Pelargonium graveolens*) against the cotton leafworm Spodoptera littoralis (Boisd.) under controlled laboratory conditions. Nano-formulations were prepared using polyethylene glycol (PEG) via oil-in-water emulsification, achieving high encapsulation efficiencies (EE%) of 96.16% for citronella and 94.16% for geranium oils, with corresponding loading capacities (LC%) of 77.43% and 76.35%, respectively. Transmission Electron Microscopy (TEM) confirmed spherical nanoparticles with diameters ranging from 20 to 60 nm. Bioassay results revealed that nano-citronella exhibited the highest insecticidal activity, with the lowest LC₅₀ value (0.4111 × 10^4^ ppm) and a toxicity index of 100, indicating approximately tenfold greater potency than its bulk counterpart. Nano-geranium showed a moderate enhancement in toxicity (LC₅₀ = 3.95 × 10^4^ ppm) compared to the bulk oil. Biochemical analyses demonstrated pronounced disruptions in enzymatic activity: chitinase activity ranged from 4.43 ± 0.94 µg NAGA/min in bulk citronella treatments to 23.70 ± 2.90 µg NAGA/min in nano-citronella. Invertase activity increased markedly, reaching 175.34 ± 3.47 µg glucose/min in nano-citronella treatments, while total protein content varied from 750.77 ± 3.85 µg/mg tissue in nano-geranium to 1315.80 ± 1.27 µg/mg tissue in bulk citronella treatments. Developmental parameters were significantly affected with nano-citronella LC₅₀ treatments, causing prolonged larval duration (19.6 ± 0.71 days) and reduced pupal weight (178.7 ± 3.18 mg) compared with control groups. Molecular docking and molecular dynamics simulations identified geraniol as a potent natural inhibitor of bacterial chitinase (PDB ID: 1CTN), forming stable π–alkyl and van der Waals interactions with Trp252, Phe373, and Ser341, and exhibiting a strong binding free energy (–20.08 kcal/mol). The geraniol–chitinase complex displayed enhanced conformational stability (RMSD = 1.15 ± 0.15 Å) and compactness (Rg = 26.9 ± 0.09 Å) relative to the unbound enzyme. Overall, these findings demonstrate that nano-encapsulation substantially enhances the insecticidal efficacy, biochemical disruption, and molecular stability of essential oils, highlighting their potential as eco-friendly, high-efficiency biopesticides within integrated pest management (IPM) programs.

**Supplementary Information:**

The online version contains supplementary material available at 10.1038/s41598-026-52470-2.

## Introduction

The cotton leaf worm, *Spodoptera littoralis* (Boisd.) (Lepidoptera: Noctuidae) is a devastating pest that causes notable economic damage to multiple crops by feeding on foliage, thus reducing plant growth and yield potential^1^. Combating insect pests is necessary for maintaining agricultural productivity and ensuring food security^2^. Safer, natural surrogates for pest control are more appealing because of growing attention about the destructive effects of chemical pesticides on the environment and human health^3^. Increased awareness regarding pesticide residues and the evolution of insect resistance has accelerated the search for eco-friendly alternatives^4^.

Essential oils have been shown to be natural pesticides^5^.. *Cymbopogon nardus* (L.)^6^ and *Pelargonium graveolens* (*Thunb.) *^7^ oils have been shown to be plant–derived pesticides , due to their insecticidal and repellent properties, attributed mainly to their high content of terpenoids such as citronellal, geraniol, and linalool^8^. However, their practical use is often limited by high volatility, poor water solubility, and rapid environmental degradation^9^. Nanotechnology offers promising solutions to overcome these limitations by improving the bioavailability, stability, and penetration of active compounds^10^ and improving the delivery of active ingredients to the target pests nano-sized particles of these oils, enhancing their effectiveness and stability and extended their residual activity^11^.

Essential oils known by their wide range of biological activities, especially they facts as antimicrobial, antifungal, antiviral, analgesic, anticarcinogenic, antiparasitic, anti-inflammatory, and antioxidant agents^12^. Citronella essential oil (CITEO) is one of the essential oils that recommended for a variety of purposes, as many studies have all agreed that citronella oil and its main ingredient geraniol are safe^13^. Essential oils are chemical substances that are easily affected by environmental factors like light, temperature. To avoid degradation, encapsulating essential oils has become a key strategy. This process not only helps maintain their chemical integrity and biological activity but also enables a controlled release into the desired medium. Such a method is crucial in preventing the rapid loss of volatile compounds in essential oils^14^. Additionally, Nanoemulsions serve as an effective delivery system for bioactive compounds sourced from plants. Therefore, Citronella and geranium emulsions can be used to improve their industrial use by decreasing the required doses^15^.

Polyethylene glycol (PEG) is a linear polymer that has chemically active hydroxyl groups at each end, making it easy to connect with various functional groups. Biomolecules or nanoparticles can be attached to linear PEG through these functional groups in a process known as PEGylation^16^. Adsorption onto nanoparticle surfaces, PEG is regularly chosen due to its properties, which include, substantial spatial repulsion, electrical neutrality and high hydrophilicity^17^. A hydrophilic protective layer is formed around the nanoparticles after PEG gelation by the mini emulsion polymerization method^16^. This study aims to evaluate the effect of *Cymbopogon nardus* (L.) and *Pelargonium graveolens* (Thunb.) oils in both bulk and nano-formulations on the cotton leaf worm *Spodoptera littoralis* under laboratory conditions.

To investigate a potential molecular mechanism for the observed insecticidal and biochemical effects (e.g., modulation of chitinase activity, molting disruption), a computational approach was employed. While the primary target of interest is insect chitinase, the high-resolution crystal structure of bacterial chitinase from *Serratia marcescens* (PDB: 1CTN) was selected as a model system for this initial study. This enzyme is a well-established reference in chitinase research due to its extensively characterized active site, which shares a conserved TIM-barrel fold and catalytic machinery with insect family 18 chitinases^18,19^. Docking against this robust model provides a proof-of-concept to assess whether the major oil constituent, Geraniol, possesses the structural compatibility to act as a chitinase inhibitor. The insights gained can inform future, more target-specific studies on insect enzymes. This search compares their impact on larval development, seeking to contribute to the advancement of sustainable pest management approaches that reduce dependence on chemical pesticides.

## Material and methods

### Essential oils

Citronella oil *Cymbopogon nardus* (L.) and Geranium oil, *Pelargonium graveolens (Thunb.)* were obtained from the oil extraction unit at the National Research Centre in Cairo, Egypt.

### Preparation of nano emulsions

Polyethylene glycol (PEG) nano emulsions were prepared using oil in water (o/w) emulsification, using modified version of the method described by^9^. The PEG- loaded nano emulsions were prepared by the mini emulsion polymerization method. Firstly, 10 g essential oil was emulsified in distilled water 1:1 (v/v) using Tween 80 by stirring for 10 min. The emulsion added drop wise to a 3% polyethylene glycol solution in a ratio of 1:1 (v/v) under continuous mechanical stirring at room temperature. The emulsion was sonicated for 30 min using ultrasonic cleaner set, model WUC-DO3H (290W, 60 Hz), and then sonicated for 3 min using a high energy ultra-sonication probe (model VCX750, 750W, 20 kHz). The loaded nano- encapsulated suspension was equilibrated overnight. Nano- emulsion were obtained as dispersion in aqueous solution.

#### Transmission electron microscopy (TEM)

The morphological shape of prepared nano-formulation was carried out using Transmission Electron Microscopy (TEM) (Jeol, JEM-2100). The nano-capsule suspension was diluted with distilled water and deposited onto a carbon coated copper grid.

#### Encapsulation efficiency (EE) and loading capacity (LC)

**Fig. 1:**
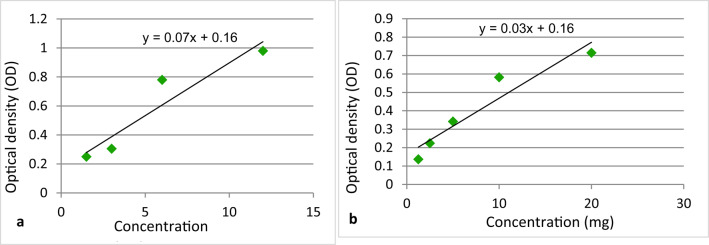
(**a**) Calibration curve of Citronella essential oil, (**b**) Calibration curve of Geranium essential oil.

Loaded nano-emulsions were evaluated for their active ingredient contents. This was carried out by refluxing of 0.5 g of loaded nano-emulsion with 10 ml of methanol at room temperature, to ensure complete extraction of encapsulated oil. The samples were then centrifuged at 10 000 g for 15 min. The supernatant layer of methanol containing the extracted oil was taken at a wavelength of 272 nm for citronella sample^20^ and 345 nm for geranium sample^21^ in an ultraviolet spectrophotometer, CHEM-7, using absolute methanol as a blank. Each sample for both oils replicated 3 times. Oil concentration was calculated by using of a calibration curve obtained from samples of each oil (Fig. 1a and b). The encapsulation parameters were determined as follows:

$$\% {\mathrm{Encapsulation}}\;{\mathrm{Efficiency}}\left( {{\mathrm{EE}}\% } \right) = \frac{{{\mathrm{The}}\;{\mathrm{amount}}\;{\mathrm{of}}\;{\mathrm{oil}}\;{\mathrm{measured}}\;{\mathrm{in}}\;{\mathrm{the}}\;{\mathrm{supernatant}}}}{{{\mathrm{Total}}\;{\mathrm{amount}}\;{\mathrm{of}}\;{\mathrm{oil}}}} \times 100$$

$$\% {\mathrm{Loading}}\;{\mathrm{capacity}}\left( {{\text{LC }}\% } \right) = \frac{{{\mathrm{The}}\;{\mathrm{amount}}\;{\mathrm{of}}\;{\mathrm{oil}}\;{\mathrm{measured}}\;{\mathrm{in}}\;{\mathrm{the}}\;{\mathrm{supernatant}}}}{{{\mathrm{Total}}\;{\mathrm{weight}}\;{\mathrm{of}}\;{\mathrm{nano}}\;{\mathrm{emulsion}}}} \times 100$$

### Insect rearing

#### Study insect

The Study insects cotton leaf worm *S. littoralis* were brought from the Department of Cotton Leaf Worm, Plant Protection Institute (PPRI) in Dokki, Giza, Egypt.

Laboratory-sensitive strain of the cotton leaf worm *S. littoralis* was bred. This strain was reared for many generations without any exposure to pesticides, and the larvae were fed on castor oil plant (*Ricinus communis* L*.*) brought from Qaha agricultural research station affiliated with the plant protection research institute. Leaves at a temperature of 27 ± 2 ◦C and a relative humidity of 65 ± 5%^22^. The eggs were kept in separate Petri dishes until they hatched. To ensure age synchronization and minimize physiological variability, only newly molted fourth instar larvae (within 6–12 h of molting) were selected for the bioassay experiments. Early larval instars (1st-3rd) were reared in groups using sterilized plastic Petri dishes (90 × 15 mm), then transferred to glass jars (1 Liter capacity, approximately 10 cm in diameter). The jars were covered with muslin cloth held by rubber bands to ensure adequate ventilation. For Pupation and Adult Emergence,

Full-grown larvae were placed in jars containing a 5 cm layer of sterilized fine sand or peat moss for pupation. Upon emergence, adults were kept in large mating jars (1–2 Liters) and provided with a 10% honey solution.

#### Bioassay experiment

The fourth larval instars of S. littoralis were reared under controlled laboratory conditions of 25–27 ± 2 °C temperature and 65 ± 5% relative humidity. Three replicates were conducted for each treatment and the control group, with ten larvae placed in each replicate, totaling 30 larvae per concentration. This sample size is consistent with established entomological bioassay protocols for preliminary screening and dose–response studies, particularly for compounds with high efficacy and clear statistical trends^23,24^. Series concentrations 10 × 103, 20 × 103 and 40 × 103 ppm were prepared using distilled water with addition of tween 80 as an emulsifier to examine the activity of citronella and geranium essential oils. The leaf-dip technique was used^25^. Fresh clean castor leaves were immersed in each tested concentration for 30 s. Then, leaves are allowed to dry at room temperature for 30 min. This duration ensured the complete evaporation of water while maintaining a uniform film of the essential oil on the leaf surface, following the standardized protocols of Khalil et al^26^. and Abdel-Sattar et al^27^.. Treated leaves were presented to 4th instar of S. littoralis larvae for 24 h, then were replaced by untreated fresh leaves. Mortality was recorded after 1, 3, 5 and 7 days. A crucial aspect of our experimental design was the inclusion of an untreated control group, where larvae were fed on leaves immersed in distilled water only. As shown in Table 3, the control group consistently exhibited 0% mortality throughout the experiment, confirming the absence of natural mortality and validating the observed effects of the essential oil treatments. Therefore, Since the control group consistently exhibited 0% mortality, Abbott’s formula for mortality correction was not required, as background mortality did not exceed the 5% threshold^28^. This confirms the high reliability of the observed mortality data.

#### Biological and developmental studies

To evaluate the sub lethal effects of tested essential oils on the biological parameters of *S. littoralis*, the following methodology was adopted:

- **Sub lethal concentration treatment:** The LC_50_ values, previously determined at 3 days post-treatment for both citronella and geranium nano-emulsions, were applied to the 4^th^ instar larvae.
- **Experimental design and sample size:** A total of 50 4^th^ instar larvae were utilized for each treatment and the control group. These were divided into five replicates, with 10 larvae per replicate, to ensure statistical accuracy in monitoring developmental transitions.
- **Application method:** The leaf-dipping technique was employed, where fresh castor bean leaves were immersed in the respective LC_50_ concentrations for 30 s and allowed to air-dry before being offered to the larvae. The control group was fed on leaves dipped in distilled water only.
- **Monitoring larval and pupal development:**Following the method described by Abdel-Sattar et al^27^., surviving larvae were monitored daily to record the duration of the larval stage and the subsequent pupation success. The pupal duration was also recorded for individuals that successfully reached the pupal stage.
- **Weight measurements**: According to the protocol of Khalil et al^26^., pupae were collected and weighed individually 24 h after pupation using a sensitive electronic balance (0.001 g accuracy) to determine the mean pupal weight for each treatment group.
- **Morphological abnormalities**: Visual inspection for any morphological deformities in larvae, pupae, and emerging adults was conducted daily. Specific focus was placed on identifying larval-pupal intermediates and adult emergence failures as indicators of growth disruption.

### Biochemical activity

#### Preparation of enzyme extracts

To assess the total protein content and enzyme activities, 4th instar larvae of *S. littoralis* were treated with LC_50_ values of nano and bulk emulsions prepared from the tested oils (Citronella and geranium). After 3 days the larvae were homogenized with sodium phosphate buffer (0.1 M, pH 7.0) at a ratio of 0.1 g of body weight per 1.00 ml of buffer. The homogenates were then centrifuged at 6,000 rpm for 15 min at 4 °C, and the supernatant served as the source of enzymes. This supernatant was collected and stored at -20˚C until assays.

#### Determination of chitinase activity

Chitinase activity was evaluated using 3, 5-dinitrosalicylic acid (DNSA) reagent to quantify the free aldehydic groups of N-acetylglucosamine released during chitin digestion Ishaaya and Casida^29^. The reaction mixture contained 0.12 ml of 0.2 M phosphate buffer at pH 6.6, 0.3 ml of 0.5% colloidal chitin, and 0.18 ml of larval homogenate. After incubating for 60 min at 37 °C, the reaction was ended by adding 1.2 ml of the DNSA reagent. The mixture was then heated for 5 min at 100 °C, cooled in an ice bath, and diluted with 1.2 ml of distilled water. To separate undigested chitin, the mixture was centrifuged for 15 min at 6000 rpm, and the absorbance of the supernatant was measured at 550 nm. A direct reaction between N-acetylglucosamine (NAGA) and the DNSA reagent, under conditions similar to those used in the enzyme reaction, produced a linear relationship between absorbance values and the amounts of NAGA. Specifically, 1 mg of NAGA resulted in an absorbance value of 0.78. The specific activity of chitinase is reported as grams of N-acetylglucosamine released per minute.

#### Determination of invertase activity

The activity of carbohydrate hydrolyzing enzyme, invertase, which breaks down sucrose, was analyzed following the methodology outlined by Ishaaya and Swirski in 1976. This technique involved the digestion of sucrose using invertase. The amount of free aldehydic group of glucose produced from the digestion of starch and/or sucrose was assessed using 3,5-dinitrosalicylic acid reagent. Enzymatic activity was quantified as µg of glucose released per minute. The reaction mixture comprised 0.2 ml of 4% sucrose (the substrate), 0.18 ml of 0.2 M acetate buffer (pH 5.4), and 20 µl of larval homogenate.

#### Preparation of standard calibration curve for glucose

To create a standard calibration curve for glucose, serial concentrations of glucose solutions containing 50, 100, 150, 200, and 250 µg of glucose in 0.4 ml of distilled water were pipetted into test tubes^30^. Each tube received 800 µl of dinitrosalicylic acid solution. These mixtures were heated for 5 min at 100 °C in a boiling water bath and then immediately cooled in an ice bath. The resulting color was measured spectrophotometrically at 550 nm. The optical densities were plotted against the concentrations to construct a curve, plotting O.D. (Optical Density) versus concentration.

#### Total protein determination

The Bradford method^31^ was utilized for protein quantification. To prepare the protein reagent, 100 mg of Coomassie Brilliant Blue G-250 was dissolved in 50 ml of 95% ethanol, followed by the addition of 100 ml of 85% (W/V) phosphoric acid. This solution was then brought up to a final volume of 1 L.

A sample solution of 50 μl, along with standard curve preparations containing serial concentrations of 700 to 2000 μg of bovine serum albumin, was placed in test tubes. The total volume in each tube was adjusted to 1 ml using the 0.1 M phosphate buffer (pH 6.6). To each test tube, 5 ml of the protein reagent was added, and the contents were mixed either by inversion or vortexing. Absorbance readings were taken at 595 nm using a UV/VIS spectrophotometer (model CHEM-7) after 2 min and within 1 h (to prevent changes in the colorimetric absorption after this period), against a blank prepared with 1 ml of phosphate buffer and 5 ml of protein reagent. The protein content was expressed as µg/mg of tissue as follows:

$$Proiein\;content\left( {\mu g/mg \;tissue} \right) = \frac{Absorbance\;of\;sample}{{Absorbance\;of\;standard}} \times conc.\;of\;standard \times \frac{1}{mg \;tissue}$$

### Calculating toxicity index and relative potency were as followed

Statistical analysis of mortality data was performed using Probit analysis. Since mortality in the control group was 0%, Abbott’s formula was not applied, ensuring that the calculated LC_50_ values directly reflect the treatment efficacy.

$${\text{Toxicity Index}} = \frac{{{\mathrm{LC}}_{{{5}0}} \;{\mathrm{value}}\;{\mathrm{of}}\;{\mathrm{the}}\;{\mathrm{highest}}\;{\mathrm{efficiency}}\;{\mathrm{formulation}}}}{{{\mathrm{LC}}_{{{5}0}} {\mathrm{value}}\;{\mathrm{of}}\;{\mathrm{the}}\;{\mathrm{other}}\;{\mathrm{formulation}}}} \times 100$$

$${\mathrm{Relative}}\;{\mathrm{potency}} = \frac{{{\mathrm{LC}}_{{{5}0}} \;{\mathrm{value}}\;{\mathrm{of}}\;{\mathrm{the}}\;{\mathrm{least}}\;{\mathrm{efficiency}}\;{\mathrm{formulation}}}}{{{\mathrm{LC}}_{{{5}0}} \;{\mathrm{value}}\;{\mathrm{of}}\;{\mathrm{the}}\;{\mathrm{other}}\;{\mathrm{formulation}}}}$$

### System preparation and molecular docking

The crystal structure of bacterial chitinase (PDB ID: 1CTN)^18^ was retrieved from the Protein Data Bank and prepared using UCSF Chimera (version 1.17; https://www.cgl.ucsf.edu/chimera/)^32,33^. Using PROPKA (version 3.1,https://github.com/jensengroup/propka)^34^, the pH was fixed and optimized to 7.5. The two-dimensional ligand structure was drawn using ChemBioDraw Ultra (version 12.1; https://revvityinformatics.com/products/ChemOffice-chemdraw) (BETHANY, 2014). The steepest descent approach and the MMFF94 force field in Avogadro software (version 1.2.0; https://avogadro.cc/) were used to optimize the 2D structure for energy minimization. Finally, hydrogen atoms were removed using UCSF Chimera^32,33^ in preparation for docking.

#### Molecular docking

Molecular docking simulations were performed using AutoDock Vina (version 1.2.0; https://vina.scripps.edu/). Prior to docking, Gasteiger partial charges were assigned to the ligand, and AutoDock atom types were defined using the graphical user interface of MGL Tools https://ccsb.scripps.edu/mgltools/^35^. The grid box was centered at coordinates (x = 24.64, y = 90.69, z = 19.04) with dimensions of 20 Å^3^ and an exhaustiveness value of 8. Conformational sampling was driven by the Lamarckian genetic algorithm, which generated docked poses ranked by their calculated binding affinity.

#### Molecular dynamic (MD) simulations

Molecular Dynamics (MD) simulations provide a powerful methodology for investigating the physical motions and temporal evolution of biological systems, offering insights into dynamic processes such as conformational changes and binding events that are difficult to capture experimentally^36^. In this study, all MD simulations were performed using the GPU-accelerated PMEMD engine within the AMBER 18 package https://ambermd.org/^37^. Initial system preparation involved assigning partial atomic charges to each compound via the General Amber Force Field (GAFF) using the ANTECHAMBER tool^38^. The LEaP module was then employed to solvate each system in an orthorhombic box of TIP3P water molecules, maintaining a 10 Å buffer, and to add Na + /Cl- counter ions for system neutralization.

The simulation protocol commenced with a two-stage energy minimization. First, 2000 steps of minimization were conducted with a 500 kcal/mol restraint on solute atoms, followed by an unrestrained minimization of 1000 steps using the conjugate gradient algorithm. Subsequently, each system was gradually heated from 0 to 300 K over 500 ps in the NVT ensemble, applying a 10 kcal/mol harmonic restraint to solute atoms. This was followed by a 500 ps equilibration period at 300 K in the NPT ensemble, with pressure maintained at 1 bar using the Berendsen barostat^39^. Production simulations were then carried out in the NPT ensemble (300 K, 1 bar) using a Langevin thermostat with a collision frequency of 1 ps⁻^1^ and a pressure-coupling constant of 2 ps. The integration time step was set to 2 fs, with all bonds involving hydrogen atoms constrained using the SHAKE algorithm. A single-precision floating-point model was utilized throughout, with randomized seeding for each simulation.

#### Post-MD analysis

Following the acquisition of trajectories from MD simulations at 1 ps intervals, the trajectories were examined utilizing the CPPTRAJ module of the AMBER18 suite. https://ambermd.org/ The OriginLab https://www.originlab.com/^40^ data analysis software and Chimera https://ambermd.org/)^32,33^ were utilized to generate all graphs and visualizations.

#### Thermodynamic calculation

The Molecular Mechanics with Generalized Born and Surface Area continuum solvation (MM/GBSA) and Poisson-Boltzmann Surface Area (MM/PBSA) methods are well-established for estimating ligand-binding affinities, providing a robust computational approach within a defined force field^41–43^. These methods yield statistically-mechanical estimates of binding free energy from molecular simulations. In this work, the binding free energy was calculated by averaging across 200 snapshots extracted from the final 20 ns of the production trajectory. The change in binding free energy (ΔG_bind) for each species—complex, ligand, and receptor—was computed according to the following formalism^44^:

The binding free energy was decomposed into gas-phase and solvation contributions. The gas-phase energy (E _gas_) comprises the internal (E _int_), electrostatic (E _ele_), and van der Waals (E _vdw_) terms, which were calculated directly from the FF14SB force field parameters. The solvation free energy (G_sol_) was determined as the sum of polar (G _GB_) and non-polar (G_SA_) components. The non-polar solvation energy was computed based on the Solvent Accessible Surface Area (SASA), employing a probe radius of 1.4 Å, while the polar solvation contribution was derived by solving the Generalized Born (GB) equation. In this formalism, S and T represent the total entropy of the solute and the absolute temperature, respectively. Furthermore, a per-residue energy decomposition was performed using the MM/GBSA methodology within Amber18 to quantify the contribution of individual residues to the total binding free energy.

### Statistical analysis

Mortality data were processed; however, Abbott’s formula^28^ was not applied in this study because the control mortality was 0%, which simplifies the analysis and enhances data reliability, and analyzed using probit regression analysis following the method of Finney^45^ to estimate the lethal concentration values (LC_50_), and their 95% confidence limits. The slope of the regression line, standard error (SE), chi-square (χ^2^) value, and regression statistics (g, h, and r) were calculated to assess the goodness-of-fit and precision of the estimates. Significance between treatments were calculated using one-way ANOVA and Tukey’s HSD test in SPSS (version 16, URL: https://www.ibm.com/products/spss-statistics ) (IBM Corp., USA), p < 0.05.

## Results and discussion

### Transmission electron microscopy (TEM)

**Fig. 2:**
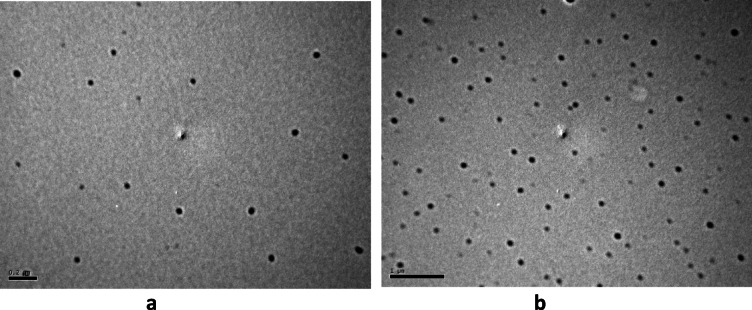
Transmission electron microscopy of prepared loaded nano-emulsions: (**a**) TEM image of nano-citronella, (**b**) TEM image of nano-geranium.

^46^ prepared citronella nano-emulsion and gained nano-particles less than 200 nm. But they used longer period of probe ultrasonic treatment (6 min, amplitude 50%). In this investigation we used shorter period of 3 min, as the longer period in general may affects the active ingredients of citronella oil, causing loss of their biological pesticidal activity. Our findings were in parallel with those obtained by^47^. They prepared nano-emulsion from citronella oil with smallest droplet size (33 nm). Also^48^, and^49^ prepared essential oils loaded in PEG ranged between 20–60 nm in diameter.

^50^ gained spherical nano particles of geranium oil emulsion with size droplet 500 nm. But they just used mechanical stirring to obtain nano emulsion without using high energy ultrasonic probe. It was clear that ultrasonic probe is necessary to obtain smaller particles. This suggestion is in agreement with^12^. They reported that mechanical stirring is not sufficient to obtain nano particles less than 100 nm in diameter.

Both nano-citronella and nano-geranium were examined by TEM in order to estimate their morphological shapes. As shown in Fig. 2 nano-citronella and nano-geranium loaded nano particles take the smooth and spherical shape. + the scale bare of the photos.

### Encapsulation efficiency (EE) and loading capacity (LC)

Encapsulation efficiency (EE%) and loading capacity (LC%) of both nano-citronella and nano-geranium loaded nano emulsions were calculated and reported in Table 1. It was shown that EE% were 96.16 and 94.16 for nano-citronella and nano-geranium, respectively. LC% were 77.43 and 76.35, respectively.

**Table 1:** Encapsulation efficiency (EE%) and Loading capacity (LC%) for citronella and geranium nano-emulsions.

Parameter	Nano-Citronella (Mean ± SD)	nano-geranium (Mean ± SD)
EE%	96.16 ± 3.34	77.43 ± 7.69
LC%	94.16 ± 3.37	76.35 ± 7.59

#### Toxicity of essential oils nano-emulsions on *S. littoralis* larvae:

The toxicity parameters summarized in Table 2 revealed significant differences between the lethal concentration (LC_50_) values of nano and bulk formulations of citronella and geranium essential oils. The LC_50_ value of nano-citronella (0.4111 × 10^4^ ppm; 95% CI: 0.2349–0.5927) was substantially lower than that of its bulk counterpart (2.09 × 10^4^ ppm; 95% CI: 1.67–2.64), indicating an increase in toxicity following nano-formulation. Accordingly, nano-citronella exhibited the highest toxicity index (100), followed by bulk citronella (19.67), nano-geranium (10.41), and bulk geranium (9.74).

**Table 2:** Toxicity parameters of nano and bulk formulated essential oils on *S. littoralis* larvae.

Essential oil	Treatment	LC50 (ppm × 10^4^) (confidence limit)	Slope	Regression	Toxicity index	Relative potency
Citronella	Nano-emulsion	0.41 (0.23–0.59)	2.28 ± 0.72	0.97	100	10.27
	Bulk oil	2.09 (1.67–2.64)	3.16 ± 0.64	0.16	19.67	2.02
Geranium	Nano-emulsion	3.95 (1.84–9.98)	1.30 ± 0.43	0.44	10.41	1.07
	Bulk oil	4.22 (3.00–10.71)	2.20 ± 0.65	0.33	9.74	1.00

Similar findings were reported by Mohamed^51^, who demonstrated that nano-formulations of geranium and garlic essential oils exerted greater toxicity against *S. littoralis* larvae than their bulk forms. This enhanced insecticidal activity can be attributed to the increased surface area and improved bioavailability of essential oils upon nano-encapsulation, which facilitates greater interaction with larval tissues and enhances larvicidal efficacy.

Our choice of concentrations (10 × 10^3^, 20 × 10^3^ and 40 × 10^3^ppm) was strategically made to investigate the long-term impact on the pest’s life cycle rather than mere immediate mortality. According to Bhagat et al^52^., nano-insecticides are specifically engineered to provide high efficacy at low application rates. This is further supported by Pavela (2018^53^) and Badawy et al. (2010^54^), who utilized similar ranges to study the growth-inhibitory effects on *S. littoralis*.

#### Cumulative mortality percentages

As shown in Table 3, cumulative mortality percentages were recorded at 1, 3, 5, and 7 days post-treatment, indicated that nano-citronella and nano-geranium exhibited significantly higher insecticidal activity compared to their bulk counterparts.

**Table 3:** Cumulative mortality percentage of the 4th instar larvae of *S. littoralis* treated with different concentrations of essential oils.

Treatment	Conc. (ppm)	Day 1 (%)	Day 3 (%)	Day 5 (%)	Day 7 (%)
Citronella nano-emulsion	10 × 10^3^	36.6	60.0	80.0	90.0
	20 × 10^3^	60.0	80.0	90.0	96.6
	40 × 10^3^	76.6	93.3	100.0	100.0
Citronella bulk oil	10 × 10^3^	16.6	26.6	40.0	53.3
	20 × 10^3^	30.0	46.6	60.0	70.0
	40 × 10^3^	46.6	66.6	80.0	90.0
Geranium nano-emulsion	10 × 10^3^	8.9	20	32.23	38.9
	20 × 10^3^	14.43	34.43	46.66	53.33
	40 × 10^3^	27.76	50.00	61.1	70
Geranium bulk oil	10 × 10^3^	1.1	7.7	17.76	28.9
	20 × 10^3^	4.43	23.33	41.1	52.33
	40 × 10^3^	14.44	46.66	65.55	78.88
Control	0%	0.0	0.0	0.0	0.0

### Developmental parameters

The developmental impacts on fourth instar *S. littoralis* larvae treated with LC_50_ concentrations of nano-formulations (0.41 × 10^4^ ppm for citronella and 3.95 × 10^4^ ppm for geranium) are detailed in Table 4. A significant prolongation was observed in nano-citronella treatment (19.67 ± 0.33 days) compared to the control (16.00 ± 0.58 days). Conversely, no significant difference was found between the nano-geranium treatment (16.67 ± 0.33 days) and the control.

**Table 4:** Developmental parameters of *S. littoralis* larvae treated with LC_50_ of nano formulations of citronella and geranium essential oils.

Treatment	Larval duration/day (mean ± SE) (lower bound–upper bound)	Pupal duration /day (mean ± SE) (lower bound–upper bound)	Pupal Weight /mg (mean ± SE) (lower bound–upper bound)
LC50 of Citronella nano-emulsion (0.41 × 104 ppm)	19.67 ± 0.33a (18.23–21.10)	11.67 ± 0.88a (7.87–15.46)	178.7 ± 3.18c (165.0–192.3)
LC50 of Geranium nano-emulsion (3.95 × 104 ppm)	16.67 ± 0.33b (15.23–18.10)	10.67 ± 0.67a (7.80–13.54)	195.3 ± 3.32b (181.0–209.6)
Control	16.00 ± 0.58b (13.25–18-48)	10.00 ± 0.58a (7.52–12.48)	220.4 ± 0.72a (217.3–223.5)
F-value	20.60**	1.36 NS	61.09**

Values are expressed as Mean ± SE. Different letters indicate significant differences between treatments at *P* < 0.05 using Tukey’s HSD test

NS, not significant

**highly significant

​Pupal duration: No significant differences were recorded across treatments, with durations of 11.67 ± 0.88 days for nano-citronella, 10.67 ± 0.67 days for nano-geranium, and 10.00 ± 0.58 days for control.

​Pupal weight: Both treatments caused a significant reduction in pupal weight (178.7 ± 3.18 mg for nano-citronella and 195.3 ± 3.32 mg for nano-geranium, relative to the control (220.4 ± 0.72 mg).

The present study demonstrated that nano-formulations of citronella and geranium oils exerted notable sub lethal effects on *S. littoralis* when applied at LC_50_ concentrations. These effects were evident in larval development and pupal weight, indicating disruption of normal growth and metamorphosis.

The significant prolongation of larval duration observed in nano-citronella treated larvae suggests interference with normal physiological and hormonal regulation of development. Prolonged larval periods are often associated with impaired feeding efficiency, metabolic stress, or disruption of ecdysteroid and juvenile hormone balance, which delays molting and development. Similar developmental delays have been reported in insects exposed to botanical insecticides and essential oil-based formulations^55^, supporting the hypothesis that citronella nano-formulation may act as a growth regulator in addition to its toxic effects. In contrast, the absence of a significant effect on larval duration in geranium nano-treated larvae indicates a comparatively weaker influence on developmental timing, despite its toxic action. These results are consistent with Mostafa et al^56^., who found that citronella oil slightly prolongs larval and pupal duration in *S. littoralis* at LC_15_ and LC_50_ doses. While direct data for geranium oil on *S. littoralis* is limited, it is known to reduce pupal weight in related lepidopteran species. Such sub-lethal effects successfully disrupt insect development without necessarily altering the total duration of the pupation period.

Pupal duration was not significantly affected by either nano-formulation, suggesting that once larvae successfully pupated, the metamorphic process proceeded at a relatively normal rate. This may indicate that the major impact of the nano-formulations occurs during the larval stage rather than during pupal development.

The significant reduction in pupal weight observed in both nano-treated groups compared with the control reflects compromised larval growth and nutrient assimilation. Reduced pupal weight is commonly associated with decreased larval feeding, impaired digestion, or increased energy expenditure for detoxification processes^55^. Such reductions are biologically important, as pupal weight is directly correlated with adult fitness, fecundity, and survival. The more pronounced reduction in pupal weight in citronella nano-treated insects further supports its stronger physiological impact compared with geranium nano-formulation.

Overall, the results indicate that citronella and geranium nano-formulations induce sublethal developmental effects in *S. littoralis*, with citronella showing greater potential to disrupt larval growth and development. These findings highlight the potential of essential oil-based nano-formulations as eco-friendly alternatives to conventional insecticides, capable of reducing pest fitness even at sublethal concentrations.

The appearance of larval-pupal intermediates and the failure of adult emergence suggest a profound interference with the insect’s endocrine system. According to Khalil et al^26^., nano-formulations of essential oils act as growth regulators that disrupt the balance between juvenile hormone and ecdysone. ​The primary constituents of citronella and geranium oils-namely citronellal, geraniol, and citronellol induce toxicity in *S. littoralis*larvae through multiple mechanisms. As noted by Khalil et al^1^., these compounds disrupt midgut epithelial cells, inhibit digestive enzymes (such as proteases and amylases), and trigger oxidative stress that damages vital proteins and lipids. Furthermore, Adel et al^57^. highlighted that geranium oil exhibits high potency, leading to elevated larval mortality, reduced levels of hemolymph proteins and lipids, and the suppression of detoxification enzymes like carboxylesterases. The monoterpenoids in both oils penetrate the insect cuticle to impair nutrient uptake and disrupt metabolism. Nano-formulations amplify these effects by enhancing chemical stability and cuticle penetration, leading to profound enzyme inhibition even at equivalent doses.

### Biochemical analysis

The biochemical effects of nano-formulations compared to the bulk oil were tested against 4th instars larvae of *S. littoralis*. The tested larvae were treated by the calculated LC_50_ values of each formulation prepared from citronella or geranium oil. Results of biochemical effects (i.e. Chitinase, Invertase, and Total Protein content) of nano-formulations are listed in (Table 5).

**Table 5:** Biochemical Effects of Nano and Bulk formulated Essential oils on S*. littoralis larvae.*

Treatment	Formulation	Chitinase µg NAGA/min (lower bound–upper bound)	Invertase µg glucose/min (lower bound–upper bound)	Total protein µg/mg tissue (lower bound–upper bound)
Citronella	Nano (0.41 × 10^4^ ppm)	23.70 ± 2.90a (11.22–36.18)	175.34 ± 3.47d (160.41–190.26)	774.87 ± 27.38b (657.07–892.97)
	Bulk (2.09 × 10^4^ ppm)	4.43 ± 0.94c (0.39–8.47)	426.98 ± 24.49b (321.58–532.37)	1315.80 ± 1.27a (768.30–1863.23)
Geranium	Nano (3.95 × 10^4^ ppm)	15.10 ± 1.38b (9.18–21.03)	285.82 ± 16.80c (213.52–358.12)	750.77 ± 3.85b (734.22–767.32)
	Bulk (4.22 × 10^4^ ppm)	25.37 ± 2.93a (12.79–37.99)	261.12 ± 34.67 cd (111.95–410.29)	769.74 ± 67.03b (481.33–1058.15)
Control	–	25.52 ± 2.60a (14.32–36.73)	582.84 ± 40.19a (409.89.755.78)	1269.60 ± 61.95a (1003.05–1536.18)
F value		15.65**	34.55**	16.58**

**indicates highly significant difference (*p* < 0.05)

Values are expressed as Mean ± SE. Different letters in the same column means significant differences between treatments, using Tukey’s HSD test.

#### Chitinase activity

**Fig. 3:**
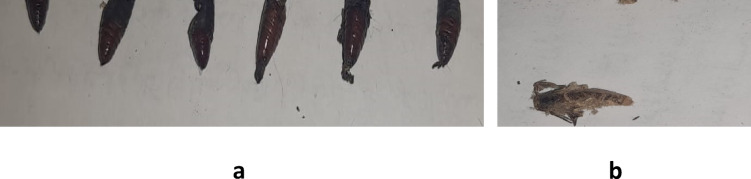
Malformations resulted from Nano-emulsions treatments. (**a**) Malformations in pupae, (**b**) Malformations in adults.

The lowest value of chitinase activity was observed with Citronella Bulk oil (4.43 ± 0.94 µg NAGA/min), while the Control was the highest (25.52 ± 2.60 µg NAGA/min), followed by geranium nano-emulsion (15.10 ± 1.38 µg NAGA/min). Bulk geranium was (25.37 ± 2.93 µg NAGA/min). As shown in Table 4, bulk citronella and nano-geranium treatments exhibited significant suppression to chitinase activity. These findings can be explained as pure Citronella oil’s active compounds may penetrate larval cuticles rapidly to inhibit chitinase, essential for molting in fourth instar stages. Nano-formulations create a controlled release matrix, which sustains overall lethality but fails to match pure oil’s acute enzyme suppression, possibly due to slower diffusion at target sites. This explains the statistically significant efficacy gain in nano-citronella for mortality, yet no chitinase difference from controls. Geranium oil provides consistent chitinase inhibition regardless of formulation, as its less volatile nature allows nanoencapsulation to mimic free oil delivery without significant alteration. No efficacy difference arises because the nanoform does not hinder penetration, maintaining equivalent enzyme disruption and larval mortality. This stability contrasts Citronella’s volatility, where nano-trapping reduces peak bioactive concentrations at enzymes. Malformation symptoms related to enzymes activity disturbance were stated in Fig. 3a and b.

#### Invertase activity

The highest activity of invertase activity was with citronella loaded nano-emulsion (175.34 ± 3.47 µg glucose/min), while the control was higher (582.84 ± 40.19 µg glucose/min) followed by bulk geranium (261.12 ± 34.67 µg glucose/min), the lowest activities were observed with bulk citronella (426.98 ± 24.49 µg glucose/min). Generally, all treatments did not exceed the control, nano-citronella significantly increased invertase activity compared to other treatments. Conventional oils exhibited intermediate effects. Essential oil compounds penetrate insect midgut cells, inhibiting invertase a key enzyme that hydrolyzes sucrose into glucose and fructose for energy leading to impaired nutrient absorption and starvation effects. Nanoencapsulation enhances this by improving oil stability, solubility, and controlled release, allowing deeper penetration and prolonged exposure without rapid degradation. The lack of significant difference between essential oil and nano-geranium oil suggests equivalent effective doses of active compounds reached the target site, as nanoencapsulation primarily boosts bioavailability but may not exceed free oil efficacy at matched concentrations in this setup. Geranium oil’s specific monoterpenes likely saturate the inhibitory pathway similarly in both forms^58^.

#### Total protein contents

The highest protein content was in bulk citronella (1315.80 ± 1.27 µg/mg tissue) and control larvae (1269.60 ± 61.95 µg/mg tissue). While the lowest was observed in nano-geranium (750.77 ± 3.85 µg/mg tissue). It was shown that all treatments affected the total protein contents differently, with bulk treatments generally showing higher values, although nano-citronella was an exception. The lower total protein level in insects treated with essential oils and their nanoformulations usually indicates general physiological stress and tissue damage, especially in the gut and fat body where many proteins are synthesized. Essential oils contain reactive terpenoids and phenolic compounds that can disrupt cell membranes, denature proteins, and interfere with ribosomes, which reduces protein synthesis and can accelerate protein breakdown through proteolytic enzymes^59^.

All parameters (Chitinase, Invertase, Protein) showed highly significant differences (p < 0.05), indicating that the effects of treatment type and formulation are statistically significant. Nano formulations of essential oil particularly nano-citronella were more effective in disrupting metabolic and structural enzymatic systems in *S. littoralis*, likely due to improved bioavailability and cellular penetration.

Recent studies reinforce that nanoformulations of essential oils, including citronella, markedly enhance insecticidal activity and induce stronger biochemical disruptions in *S. littoralis*larvae compared to conventional bulk oils. For instance, Khalil et al^1^. found that acetylcholinesterase (AChE) activity significantly increased in both bulk and nanoemulsion forms of citronella treatment, while GST decreased and αesterase activity in fourth instar larvae; comparably, nanoform appears to have more effects than the bulk form. Adel et al^57^. found that citronella nanoformulation scored higher mortality rates and enhanced stability compared with bulk citronella oil. The present study explains that essential oils of *C. winterianus* (citronella) and *P. graveolens* (geranium), particularly in their nanoformulated forms, change the activity of main metabolic enzymes such as chitinase, invertase, and total protein levels, so exert significant biochemical effects on *S. littoralis*larvae. These results highlight the enhanced insecticidal efficacy of nanoemulsions compared to their conventional oil counterparts. The significant elevation of chitinase activity in larvae treated with nano-granium and bulk citronella oil appears to cause the instant collapse of the insect cuticle, leading to molting defects or cuticular disruption. Roy et al^8^. found that Chitinase is a crucial enzyme involved in the degradation of chitin, the major component of the insect exoskeleton, and affects molting processes, and plays an important function in insect defense as a defensive response by the insect against overexpression by phytochemicals to compensate for cellular damage caused by citronellal and geraniol. This finding is consistent with those of obtained by Regnault-Roger et al^60^., who reported the ability of terpenoids to modulate insect molting enzymes. Which may reflect an immune-compensatory response in the larvae to the phytochemical stress. Invertase plays a key role in sucrose hydrolysis, and its increased activity may be a compensatory mechanism to maintain energy balance under stress conditions induced by essential oil toxicity. CITNE-PEG and GERNE-PEG oils increased invertase activity, which may cause a disruption in carbohydrate metabolism. According to Tripathi et al. (2005^61^), essential oils rich in monoterpenes such as citronellol and linalool can interfere with energy metabolism and sugar transport in insects. Similarly, the elevated levels of total protein in treated larvae, particularly those exposed to nanoformulations, may return enhanced metabolic activity or stress-induced synthesis of detoxification and repair-related proteins. These results align with findings from Bakkali et al^62^., who reported that essential oils could induce oxidative and cellular stress responses in insects, leading to increased protein synthesis as part of a defensive reaction. Importantly, the superior performance of nanoformulated oils over their conventional counterparts can be attributed to improved bioavailability, smaller droplet size, and increased surface area, which facilitate enhanced penetration through the insect cuticle and more effective interaction with intracellular targets. Benelli et al^63^. also agreed that citronella oil nanoform significantly increases insecticidal efficacy compared to bulk oil. This observation, which is supported by several studies, indicated the role of nanotechnology in improving pesticide delivery and efficacy, as confirmed by significantly lower LC_50_values and higher toxicity indices, which allocated enhancing nano-emulsions, physicochemical properties, which improve penetration, dispersion, and bioavailability of active compounds into the insect cuticle, Ahmed et al^64^.. Also, nano-carriers protect the volatile oil constituents from rapid degradation and evaporation, ensuring sustained release and prolonged efficacy Patel, Kumar, & Verma, 2020^65^. Geranium oil formulations observed a moderate difference, possibly due to variances in chemical composition. Granium oil nano-formulation reported limited changes in efficacy upon insecticidal activity^5,66^, consistent with our findings. Nanoformulated botanical insecticides provide longer-lasting protection and authorize lower dosages, so by reducing environmental contamination, they present promising alternatives in integrated pest management (IPM) (Bergonzi & Gallori 2014^67^). The increased potency of nano citronella oil demonstrated in this study could reduce the frequency of applications and enhance safety profiles, offering significant advantages over conventional bulk formulations. Citronella and Geranium LC50 doses provide the larval period and reduce pupal weight compared to the control. The significant extent of larval duration under citronella treatment is consistent with findings by Pavela (2009)^68^, who found that essential oil compounds can interfere with larval development by disrupting endocrine regulation of molting and metamorphosis. Tripathi et al. (2014^69^) noted that sub-lethal doses of botanical insecticides can impair growth and reduce biomass accumulation during metamorphosis. This agrees with the substantial reduction in pupal weight under both LC_50_ of citronella and granium treatments. Isman^70^evaluated the effects of essential oils, lower pupal weights, and prolonged development, which were commonly associated with increased metabolic stress and detoxification processes^5^. found that compared to the control, consistently producing the heaviest pupae, pupal weight significantly differed in all treatments and groups. Such reductions in pupal weight may have important ecological implications, potentially translating to decreased adult fitness and fecundity. Overall, botanically derived insecticides, especially those rich in terpenoids.

### Molecular dynamic and system stability

**Fig. 4:**
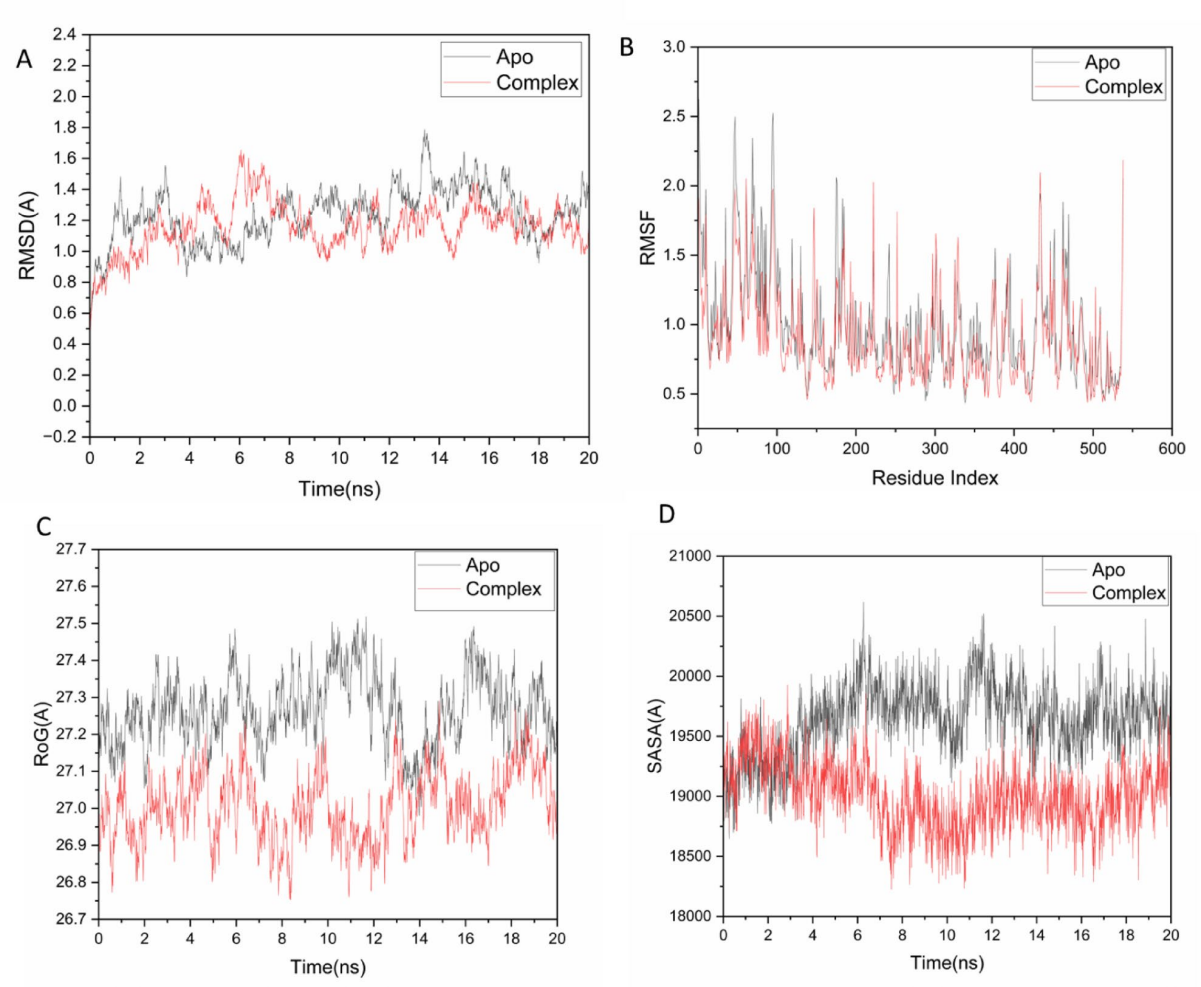
(**a**) RMSD of Cα atoms of the protein backbone atoms. (**b**) RMSF of each residue of the protein backbone Cα atoms of protein residues (**c**) ROG of Cα atoms of protein residues; (**d**) solvent accessible surface area (SASA) of the C α of the backbone atoms relative (black) to the starting minimized over 50 ns for the catalytic binding site with Geraniol – chitinase complex system (red).

Molecular dynamics (MD) simulations were employed to evaluate the potential binding efficacy and stability of Geraniol within the catalytic site of a model chitinase (1CTN). Assessing system stability is critical for identifying genuine biomolecular motions and mitigating simulation artifacts. To this end, the Root-Mean-Square Deviation (RMSD) was calculated to quantify the structural stability of the simulated systems. The average RMSD values for the apo-protein and the Geraniol-chitinase complex were 1.26 ± 0.18 Å and 1.15 ± 0.15 Å, respectively (Fig. 4A). These results indicate that the Geraniol-bound complex adopted a more stable conformation than the unbound protein.

Protein flexibility upon ligand binding was analyzed using the Root-Mean-Square Fluctuation (RMSF) to examine per-residue dynamics and their interactions with the ligand^71^. The average RMSF values for the apo-protein and the Geraniol complex were 0.98 ± 0.38 Å and 0.89 ± 0.32 Å, respectively (Fig. 4B), demonstrating that ligand binding reduced residue-level fluctuations.

The Radius of Gyration (Rg) was measured to assess the overall compactness and structural stability of the systems^72,73^. The mean Rg values for the apo-protein and the Geraniol complex were 27.26 ± 0.09 Å and 26.9 ± 0.09 Å, respectively (Fig. 4C). This suggests that the Geraniol-bound complex possesses a more rigid and compact structure.

Finally, the Solvent Accessible Surface Area (SASA) was quantified to evaluate the packing density of the protein’s hydrophobic core, a key factor in biomolecular stability^74^. The average SASA values were 19,645.88 Å^2^ for the apo-protein and 19,029.29 Å^2^ for the Geraniol complex (Fig. 4D). Collectively, the convergence of the RMSD, RMSF, Rg, and SASA analyses confirms that the Geraniol complex remains stably bound within the catalytic site of the target receptor.

The stable binding of Geraniol to the model bacterial chitinase active site, characterized by favorable van der Waals interactions and key pi-alkyl bonds, provides a plausible molecular hypothesis for the insecticidal effects observed in vivo. The conserved nature of the chitinase catalytic domain suggests that Geraniol could similarly interact with insect chitinase, potentially inhibiting its function. This aligns with our biochemical data, where treatments significantly modulated chitinase activity in *S. littoralis* larvae (Table 6), and with the life table data showing extended larval duration a hallmark of molting disruption. Therefore, while the docking target is bacterial, the results support the proposition that chitinase inhibition is a likely mechanism contributing to the toxicity of citronella and geranium oils.

**Table 6:** Shows the calculated energy binding for the Geraniol compound against the catalytic binding site of target receptor .

Complex	ΔE_vdW_	ΔE_elec_	ΔG_gas_	ΔG_solv_	ΔG_bind_
*Energy components (kcal/mol)*					
Geraniol–chitinase complex	-19.38 ± 0.55	-9.06 ± 0.47	-38.54 ± 0.59	18.36 ± 0.47	-20.08 ± 0.46

#### Binding interaction mechanism based on binding free energy calculation

The Molecular Mechanics Generalized Born Surface Area (MM/GBSA) method is a widely adopted computational technique for estimating the binding free energies of small molecules to biological macromolecules, often providing a more reliable assessment than docking scores alone. Utilizing the MM-GBSA module in AMBER18, the binding free energy was calculated by analyzing snapshots extracted from the molecular dynamics trajectories. As summarized in Table 6, all computed energy components, with the exception of the solvation free energy (ΔGsolv), exhibited strongly negative values, indicating favorable contributions to the overall binding affinity.

∆EvdW, van der Waals energy; ∆Eele, electrostatic energy; ∆Gsolv, solvation free energy; ∆Gbind, calculated total binding free energy

The interactions between Geraniol compound and the target protein receptor residues are driven by the more positive Vander Waals energy component, as shown by a detailed examination of each individual energy contribution, leading to the reported binding free energies. (Table 6).

#### Identification of the critical residues responsible for ligands binding

**Fig. 5:**
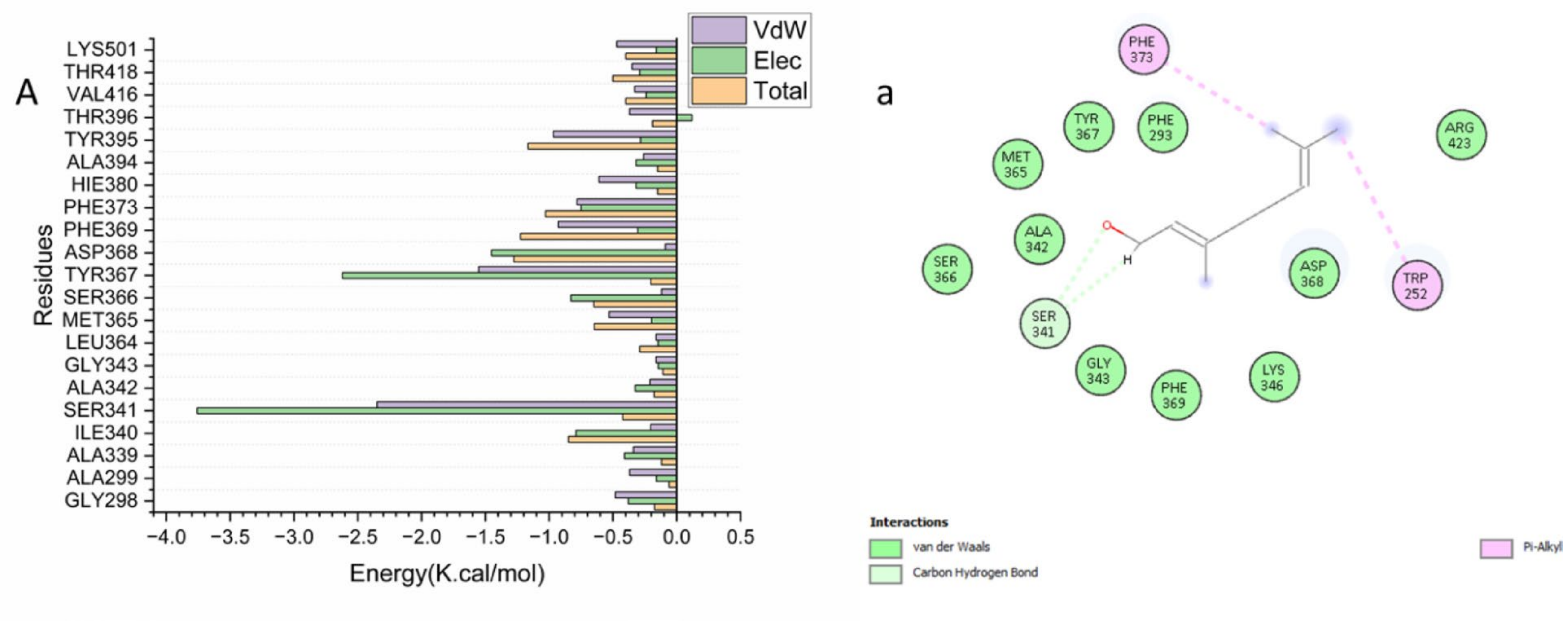
Per-residue decomposition plots showing the energy contributions to the binding and stabilization of Geraniol into catalytic binding site of chitinase receptor [A] .Corresponding inter-molecular interactions are shown [a].

A per-residue energy decomposition analysis was performed to delineate the specific contributions of active site residues to the total binding energy, thereby identifying key residues involved in the inhibition of the chitinase receptor. As illustrated in Fig. 5, the most significant favorable contributions to Geraniol’s binding within the catalytic site originate from residues Ser 341 (-2.349 kcal/mol), Tyr 367 (-1.552 kcal/mol), and Phe 369 (-0.928 kcal/mol).

#### Ligand–residue interaction network profiles

**Fig. 6:**
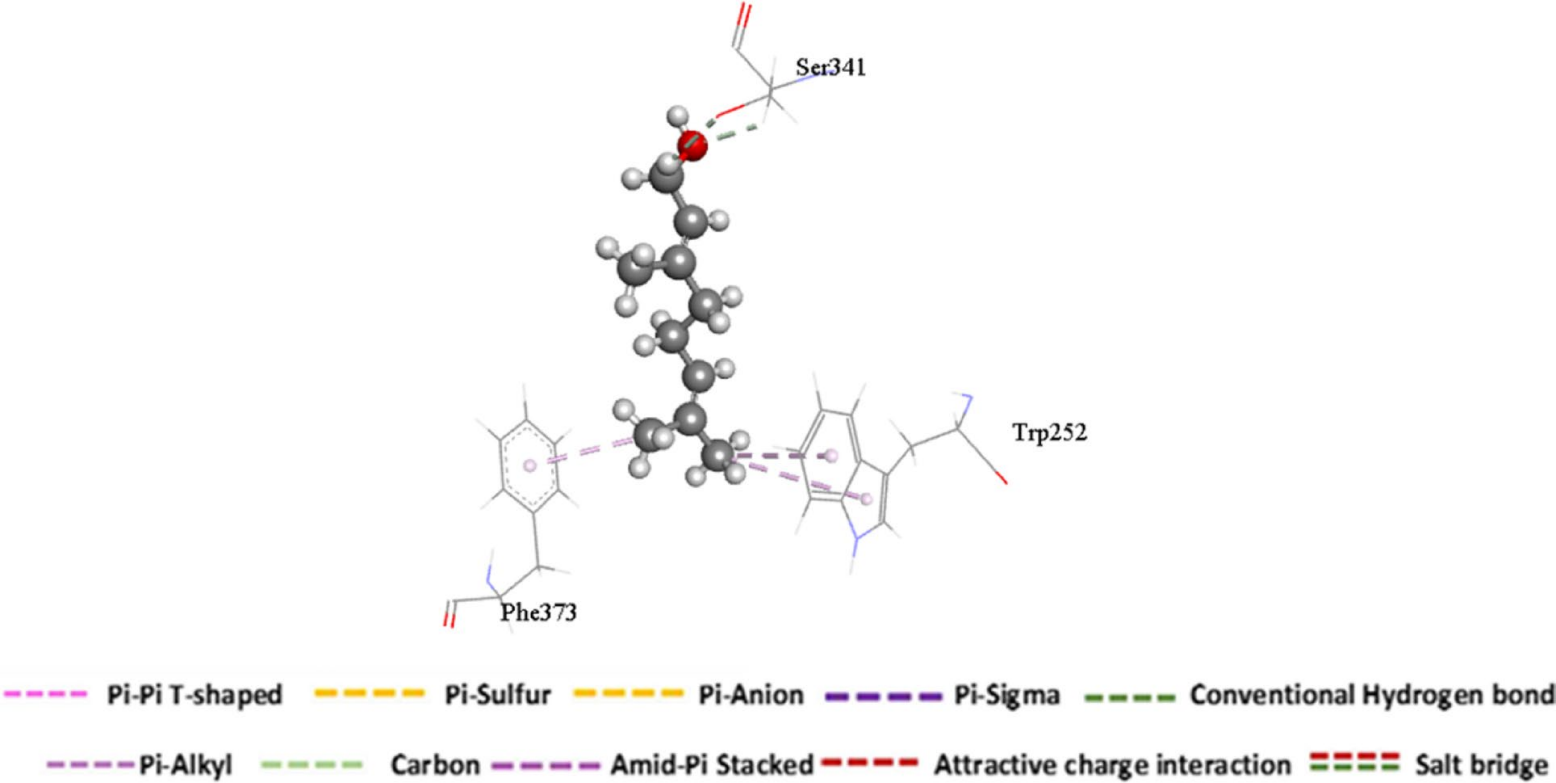
The interaction residue of Geraniol into the catalytic binding site of chitinase receptor receptor.

A fundamental objective in drug design is the structural optimization of therapeutic compounds to enhance bioavailability, mitigate toxicity, and improve pharmacokinetic profiles^75^. Analysis of the Geraniol-chitinase interaction revealed that Geraniol forms key stabilizing contacts within the binding site, including pi-alkyl interactions with residues Trp 252 and Phe 373, as well as a van der Waals interaction with Ser 341 (Fig. 6). This study identifies Geraniol as a promising candidate for chitinase inhibition. Computational analysis using a model bacterial chitinase revealed it binds stably in the enzyme’s conserved active site with strong affinity of -20.08 kcal/mol, driven primarily by van der Waals and key pi-alkyl interactions. These results provide an initial atomic-level framework for understanding the bioactivity of essential oils and inform the design of optimized inhibitors. A limitation of this study is the use of a bacterial homolog for docking; future work should employ homology modeling of *S. littoralis* chitinase to confirm target specificity and refine the mechanism.

## Conclusion

This study confirmed the high insecticidal efficacy of essential oils, especially when encapsulated into nano-formulations, against the cotton leaf worm, *S. littoralis*. Nano-formulated citronella oil {CITNE-PEG} exhibited the highest toxicity, demonstrating a tenfold increase in potency compared to its bulk counterpart. Biochemical analysis showed that nano-formulations significantly modulated key metabolic enzymes, leading to a decrease in total protein and affecting chitinase and invertase activities, suggesting disruption of the insect’s cuticle integrity and carbohydrate metabolism. These effects translated into developmental impairment, characterized by prolonged larval duration and reduced pupal weight. The molecular docking and dynamics simulations provide a structural basis for these findings, revealing that Geraniol, a key component, binds strongly to bacterial chitinase (PDB ID: 1CTN) with a favorable binding free energy, suggesting a potent mechanism of action as a chitinase inhibitor. Overall, the encapsulation of essential oils using PEG significantly enhances their stability and bioactivity, positioning them as eco-friendly alternatives for sustainable integrated pest management (IPM) strategies.

## Supplementary Information

Below is the link to the electronic supplementary material.

Supplementary Material 1

## Data Availability

All data are available in the article and the materials used in this work are of high quality and grade.
