# Supplementary material for: Comparative insecticidal efficacy and biochemical impact of nano-encapsulated citronella and geranium essential oils against Spodoptera littoralis (Lepidoptera: Noctuidae)
Source: Sci Rep. 2026 May 19;16:15475. doi: 10.1038/s41598-026-52470-2 (PMC13186964; doi:10.1038/s41598-026-52470-2)
Supplement: Supplementary file 1 — Supplementary Material 1 [file 41598_2026_52470_MOESM1_ESM.docx]

**Supplementary file**

**Table 7:** Autodocking Vina docking results for Extracted compounds docked into the catalytic domain binding site of bacterial chitinase receptor;

| Compounds | Hydrogen bonds between atoms of compounds and amino acids of receptor | | | |  | S- score  (binding energy) (kcal/mol) |
| --- | --- | --- | --- | --- | --- | --- |
|  | Compounds | receptor | | **Type** | **Distance (Å)** |  |
|  | **Atoms** | **Atoms** | **Residues** |  |  |  |
| Citronellal | O 8137 | NZ 5259 | Lys 369 | H-acceptor | 2.92 | -7.95 |
| Citronellol | H8157 | OD5610 | Asp 391 | H-donor | 2.06 | -8.68 |
| Geraniol | H 8149 | O5555 | Met 388 | H-donor | 2.06 | -9.59 |
|  | O 8135 | N 5190 | ALA 365 | H-acceptor | 2.73 |  |
|  | H4638 | O2722 | Asp1040 | H-donor | 2.19 |  |
| Limonene | No interaction |  |  |  |  |  |
